# Perceived abortion stigma and psychological well-being over five years after receiving or being denied an abortion

**DOI:** 10.1371/journal.pone.0226417

**Published:** 2020-01-29

**Authors:** M. Antonia Biggs, Katherine Brown, Diana Greene Foster

**Affiliations:** 1 Advancing New Standards in Reproductive Health (ANSIRH), Bixby Center for Global Reproductive Health, Department of Obstetrics, Gynecology and Reproductive Sciences, University of California-San Francisco, Oakland, California, United States of America; 2 Department of Obstetrics, Gynecology and Reproductive Sciences, University of California-San Francisco, San Francisco, California, United States of America; Emory University School of Public Health, UNITED STATES

## Abstract

**Objective:**

To prospectively assess perceptions of abortion stigma after receiving or being denied an abortion over 5 years, the factors associated with perceived abortion stigma, and the effects of perceived abortion stigma on psychological well-being.

**Methods:**

We recruited people seeking abortion from 30 facilities across the US, and interviewed them by phone one week post-abortion seeking, then semiannually for 5 years. We used adjusted mixed effects regression analyses to examine the abortion stigma trajectories of those who obtained an abortion near a facility’s gestational age limit (*Near-limits*) compared to those denied an abortion because they were just over the limit and carried their pregnancies to term *(Turnaway-births*).

**Results:**

Of the 956 people recruited, we removed 28 due to ineligibility or missing data, leaving a final sample of 928. In unadjusted analyses, at one-week post-abortion seeking, over half of those seeking abortion perceived that if others knew they had sought an abortion, they would be looked down upon at least “a little bit” by people close to them (60%) or by people in their community (56%). In longitudinal adjusted analyses, people denied an abortion and who carried their pregnancies to term (*Turnaway-birth* group) reported significantly lower baseline perceived abortion stigma from people close to them (-0.38; 95% CI, -0.59, -0.16) and from people in their community (0.30; 95% CI, -0.52, -0.08), than *Near-limits*, differences that remained statistically significant for 1.5 years. Overall perceived abortion stigma declined significantly (p < .001) for both study groups. High perceived abortion stigma at baseline was associated with higher odds of experiencing psychological distress years later (adjusted Odds Ratio, 3.98; 95% CI, 1.39, 11.37).

**Conclusions:**

Most people considering abortion perceive some abortion stigma, which is associated with psychological distress years later.

## Introduction

Abortion stigma is one of a broader range of reproductive stigmas that affects most people seeking abortion [1, 2]. A national study of over 4,000 U.S. abortion patients indicated that nearly two-thirds reported that people would look down on them if they knew they had an abortion [3]. Kumar, Hessini and Mitchell have defined abortion stigma as “a negative attribute ascribed to women who seek to terminate a pregnancy that marks them, internally or externally, as inferior to ideals of womanhood” [4]. By this definition, abortion stigma is specific to women seeking an abortion, whereby they feel devalued for not meeting societal expectations of motherhood. Others have conceptualized abortion stigma more broadly to also include providers and other individuals involved in abortion [5, 6]. Findings from cross-sectional studies have linked perceptions of abortion stigma with pre-abortion [7] and post-abortion psychological health [8].

Stigma is a complex concept that involves a number of interrelated factors. Pescosolido and Martin conceptualize stigma as a system or “stigma complex” which they define as “the set of interrelated, heterogeneous system structures, from the individual to the society,… that constructs, labels, and translates difference into marks. In turn, reactions … to a cultural bundle of prejudice… and discrimination… are produced” [9]. Thus, according to this and other definitions, abortion stigma is a product of the larger social, cultural and community contexts in which abortion seeking occurs [6, 10–12]. The contextual factors that define one’s community—race, ethnicity, religion, religiosity, class, age, and social support—are known to be associated with perceived abortion stigma, although the relationships are interrelated and complex [3, 6, 9, 13]. The experience of reproduction, including abortion and abortion stigma, differ depending on one’s group identity and community context [1, 9, 14]. In the U.S., a long history of White supremacy, racism and social inequalities based on race and class likely lead to differences in perceptions and experiences of abortion stigma. The largest U.S. study examining abortion stigma in the United States demonstrated that across ethnic/racial groups, African American/Black women reported lower levels of perceived and internalized abortion stigma, White women scored highest on perceptions of stigma from the general public, and Latina women scored highest in perceptions of stigma from family and friends [3]. Another national study surveying women seeking reproductive health services found that religiosity was strongly associated with overall perceived abortion stigma, but race was not [15]. However, when further examining relationships among different dimensions of stigma, African American/Black women scored lower on worries about judgment and higher on feelings of isolation, when compared to White women [15]. Being raised in a family with anti-abortion attitudes and lack of partner support have also been found to be associated with greater perceptions of abortion stigma [3, 13]. In this study, we explore the longitudinal relationships between these contextual factors, including race/ethnicity, religion and social support, on perceived abortion stigma and psychological well-being.

While perceived abortion stigma refers to people’s perceptions of how others judge them for seeking or obtaining an abortion, internalized stigma is when these views have been incorporated into one’s sense of self in the form of shame, guilt, or secrecy. People who have had an abortion and hold anti-abortion attitudes may have internalized stigma. Several participants in one qualitative study in the U.S. who had sought an abortion described anti-abortion attitudes [16]. Similarly, some people seeking abortion in England and Wales described negative views about abortion and believed they had done something morally wrong, views that were accompanied by feelings of guilt and shame [17]. While participants attributed their feelings of shame to their rejection of motherhood, those who resisted abortion stigma, justified their abortions by claiming they were being responsible mothers, in that they chose abortion in order to focus on their existing children. Participants in both studies reconciled their internalized anti-abortion attitudes with their desire to have an abortion by distinguishing their personal circumstances around their abortions from those of other people’s abortions [16, 17].

People may conceal their abortions in order to cope with their negative emotions around the abortion or to prevent negative reactions from others [3, 15, 18, 19]. A small qualitative study of women who sought an abortion in the UK, found that all women described the abortion as socially unacceptable, which motived their desire to keep it secret [19]. A national study that analyzed a subsample of women and men in the United States who had a personal experience with abortion found that over one-quarter of abortion disclosures received a negative, stigmatizing reaction [20]. However, the real-world figure is likely higher, as the study only captured disclosures among those willing to disclose their abortions as part of the study, an inherent challenge in studying abortion, as stigmatized events are notoriously underreported [21]. Similarly, several respondents in a qualitative study that specifically sampled people who had emotional difficulties related to the abortion reported that their friends and family were not supportive and some even lost relationships due to the abortion [22].

On the other hand, a substantial body of evidence outside the field of abortion indicates that concealing a stigmatized behavior, event or identity can have negative psychological consequences [23]. While there is ample evidence indicating that women keep their abortions secret [4, 5], research on the psychological effects of this secrecy is scarce. Major and Gramzow (1999) examined the effects of stigma and secrecy on experiencing intrusive thoughts and psychological distress [24]. They found that perceived abortion stigma was associated with keeping the abortion secret, that secrecy was associated with increases in thought suppression and intrusive thoughts about the abortion, and that both experiencing intrusive thoughts and thought suppression were associated with greater psychological distress. Due to simultaneous measurement of factors, all at two years post-abortion seeking, they were unable to establish causality or the sequential nature of these relationships. Findings from qualitative studies suggest that secret keeping can be experienced as a psychological burden and contribute to anxiety [19, 22]. This study will explore the relationship between abortion disclosure at the time of seeking an abortion and psychological distress up to five years later.

Enacted stigma refers to experiences of being negatively judged by family, friends, the community, or culture, whereas structural stigma, refers to discrimination from governments, organizations, institutions, and/or policies [9]. Primary examples of structural stigma in abortion care include the myriad of state-level policies that intentionally restrict people’s access to care, whereas enacted stigma may come in the form of judgment from health care providers, anti-abortion clinic protesters, and loved ones. Both enacted and structural abortion stigma can contribute to the process of stigmatization, which is the social process by which people are affected by stigma [9]. Thus, people’s interactions with medical institutions, health care providers, and policies may further contribute to their perceptions of abortion stigma and the process of stigmatization [6, 13, 19, 25]. However, these experiences are not necessarily stigmatizing for all people. Beynon-Jones (2017) described how some people navigate experiences of enacted abortion stigma by critiquing or rejecting judgment from others if they felt they were infringing on an individual’s experience [26]. Whereas, a qualitative study of people accessing abortion in the U.S. found that the presence of clinic protesters, elaborate clinic security, and the formality of clinic procedures contributed to perceived stigma [27]. Research on abortion stigma up to date, has primarily relied on cross-sectional studies, which has impeded our ability to adequately capture the social process of stigmatization.

The current U.S. political climate that has led to decreased access to abortion care represents a form of structural stigma for people needing care. Some people may experience these legal and clinic restrictions to abortion care as enacted stigma, since they may result in people feeling that they are not morally deserving of an abortion. Using Turnaway Study data, this study seeks to explore whether abortion denial due to facility gestational age limits is associated with perceived abortion stigma at the time of abortion seeking. The Turnaway Study, a longitudinal study that followed four groups of nearly 1,000 people seeking abortion, many of whom were denied the service, found evidence of internalized stigma. Approximately one in five people seeking abortion thought that abortion was morally wrong or should be illegal [28]. At six months post-abortion seeking, those denied an abortion and who carried to term (*Turnaway-births)* were the least likely to support the legal right to abortion, followed by those who obtained a *Near-limit* or *First-trimester* abortion; people who were denied an abortion and later miscarried or sought an abortion elsewhere (*Turnaway-no-births)* were most likely to support the legal right to an abortion. Further, people’s perceptions of stigma were significantly associated with reporting negative emotions (regret, anger, guilt, sadness) about their abortion years later [5, 12]. Left unanswered by the Turnaway Study is whether abortion denial affects perceptions of abortion stigma, whether perceptions of abortion stigma change over time, and whether disclosing abortion-seeking and abortion denial are associated with adverse mental health outcomes. The current analysis uses Turnaway Study data to answer these questions and to specifically assess whether abortion denial and other contextual factors are associated with perceived abortion stigma over five years post-abortion seeking and whether perceived abortion stigma and telling others about abortion seeking are associated with psychological health years later. As part of this latter research question, we tested whether race/ethnicity moderates the relationship between perceived abortion stigma and psychological health. We hypothesized that the structural stigma of abortion denial, is associated with perceptions of abortion stigma, and that perceived abortion stigma and abortion denial are associated with psychological distress years later. For all analyses we were specifically interested in assessing differences between people obtaining a *Near-limit* abortion and the three other Turnaway Study groups (*Turnaway-births*, *Turnaway-no-births*, and *First-trimester* abortion).

## Materials and methods

### Ethics statement

The study, including consent procedures, was approved by the University of California, San Francisco, Committee on Human Research (original approval date: 20 December 2006; study #: 10–00527). We obtained written informed consent from all research participants.

### Study design

In this study, we use all 11 semi-annual interview waves from the Turnaway Study, a longitudinal study designed to look at the effects of receiving versus being denied an abortion on socioeconomic status, mental health, and emotional well-being. Study details have been reported elsewhere [28–31]. From January 2008 to December 2010, we recruited people seeking an abortion at 30 facilities in 21 U.S. states. We completed all data collection for this study on January 31, 2016. Recruitment criteria included anyone who was pregnant and seeking an abortion. While we did not assess gender as part of the study, we recognize that some of the study participants may hold non binary gender identities, and thus refer to the participants as “people” throughout the study.

We selected facilities with the latest gestational age limit of any other facility within 150 miles, as recruitment sites. We recruited participants into three main study groups in a 2:1:1: ratio. Study groups included: 1) *Near-limits* (n = 452), people who sought and obtained an abortion within two weeks under the facilities gestational age limit; 2) *Turnaways* (n = 231), people who sought but were denied an abortion because they were within three weeks over the facility gestational age limit, and 3) *First-trimesters* (n = 273), people who sought and obtained a first-trimester abortion. This third group served as a secondary comparison to assess whether outcomes differed for those obtaining abortions earlier vs. later in pregnancy. Because some participants in the *Turnaway* group miscarried or had an abortion elsewhere, this group was further divided into *Turnaway-births* (n = 161) and *Turnaway-no-births* (n = 70). The 15 participants who placed their babies for adoption are included in the *Turnaway-birth* group. We interviewed participants by telephone approximately one week after seeking an abortion, then every six months through five years. The structured interview asked participants about their experiences accessing abortion, including perceived abortion stigma, childbearing and physical and mental health and well-being.

### Outcomes

Our main outcome variable, *perceived abortion stigma*, was informed by a single item stigma measure developed by Major and Gramzow [24], and adapted to a context where all people had sought but not necessarily obtained an abortion. We asked participants the extent to which they agreed with the following two statements. In the past seven days including today: 1) Have you felt that you would be looked down upon by people in your community if they knew that you had sought an abortion and 2) Have you felt that you would be looked down on by people who are close to you if they knew you had sought abortion, with choices including 0 “not at all”, 1 “a little bit”, 3 “quite a bit” and 4 “extremely”. We averaged scores across both items to serve as our primary continuous outcome: *overall perceived abortion stigma*. Individual items served as secondary outcomes, *community abortion stigma* and *abortion stigma from people close to you*. *Psychological distress* also served as a secondary, dichotomous outcome, and was measured using the Brief Symptom Inventory depression and anxiety subscales [32]. Participants who scored 9 or higher on either subscale were considered distressed.

### Independent variables

To assess our primary research question, whether abortion denial is associated with perceived abortion stigma, our primary independent variables of interest included a four-part study group variable (*Near-limit*, *Turnaway-birth*, *Turnaway no-birth*, and *First-trimester*), *years* since recruitment, and *study group x years* interactions. *Near-limits* served as the reference group. The variable *years* served to assess whether *Near-limit* outcomes changed significantly over time. S*tudy group x years* assessed whether a study group’s trajectory differed significantly from that of people in the *Near-limit* group.

To assess our secondary research question, whether perceived abortion stigma or disclosing abortion seeking affect psychological distress, and whether race/ethnicity modifies the relationship between stigma and psychological distress, we included three additional primary independent variables: baseline overall *perceived abortion stigma*, as described above, *abortion disclosure*, the number of people who were told about abortion seeking other than the man involved in the pregnancy at one-week post-abortion seeking (told no one, one person, or two or more) and *race/ethnicity X perceived abortion stigma*. For this interaction term abortion stigma was categorized into none, medium (a little bit/moderately), and high (quite a bit/extremely). The *race/ethnicity X abortion stigma* interaction term served to assess whether race/ethnicity modifies the relationship between baseline perceived abortion stigma and psychological distress. For *abortion disclosure*, we excluded telling the man involved in the pregnancy about the abortion because the overwhelming majority of men (90%) had been told about the pregnancy.

### Covariates

All models adjusted for baseline demographic characteristics including age group, highest level of education completed, and marital status. Additional covariates when modeling perceived abortion stigma include factors known to be associated with abortion stigma such as self-identified race/ethnicity, pregnancy history (parity, history of abortion and difficulty deciding to have this abortion ranging from 0 “very easy” to 4 “very difficult”), and other contextual factors including religion, exposure to clinic protesters, region of residence, ever diagnosed with depression and/or anxiety by a health professional and emotional social support as measured by six items from the Multidimensional Scale of Perceived Social Support [33, 34]. To construct a categorical race/ethnicity variable we asked participants to select from a predetermined list, the group or groups they felt best represent their race and separately asked whether they are Hispanic or Latina (Yes/No). We considered all participants who identified as Hispanic or Latina as Hispanic/Latina and included those who did not solely identify as White or Black/African American in the “other” category. To model psychological distress, in addition to controlling for demographic characteristics, we included parity, difficulty deciding to have an abortion, what the man involved in the pregnancy wanted for the pregnancy, and history of depression and/or anxiety. Gestational age at the time of recruitment was not included as a model covariate because it was highly correlated with study group, by study design. All covariates were time invariant and measured at baseline, with the exception of religion which we measured at six months post-abortion seeking.

### Statistical analyses

We used mixed effects regression analyses, accounting for clustering by recruitment site, to assess differences in demographic, pregnancy and other participant characteristics by study group at baseline (Table 1). For categorical variables with more than two categories (i.e. race/ethnicity and marital status), we used an omnibus post-estimation test to accommodate multiple category associations. For multivariable analyses with perceived abortion stigma as our outcome, we used mixed effects linear regression models to assess whether perceived abortion stigma trajectories differed by study group and changed over time, and accounted for clustering by site and individual. We assessed whether trajectories were curvilinear by testing whether including quadratic terms for time improved the model fit. All of the models with stigma as an outcome required quadratic terms for time as indicated by a significant (p < .05) likelihood ratio test. To model psychological distress, we used mixed effects logistic regression analyses to predict any psychological distress from six months through five years post-abortion seeking, adjusting for baseline factors and accounting for clustering by site and individual. To assess whether race/ethnicity modifies the relationship between perceived abortion stigma and psychological distress we included an interaction term by race/ethnicity and baseline perceived stigma. To ease interpretation of the results of the interaction term used in this model, we also used a post-estimation command to generate marginal predictive values of each categorical independent variable and to assess statistically significant differences between these variables. Because the interaction term is included in this model, one should interpret the race/ethnicity main effects as from people without any baseline perceived stigma. We conducted all analyses in STATA 15.0.

**Table 1:** Characteristics of women who sought and either received or were denied abortion, at the time they sought an abortion (N = 928).

Participant characteristics at baseline	Near-limit abortion *(reference)*	Turnaway-birth	Turnaway-no-birth	First-trimester abortion
	(n = 435)	(n = 159)	(n = 70)	(n = 264)
Age, *mean (SD)*	24.9(5.8)	23.6(5.6)*	25.06(5.6)	26.1(5.7)*
Race/ethnicity				*
*White*	36%	25%	57%	42%
*Black*	29%	34%	20%	30%
*Hispanic/Latina*	20%	28%	10%	20%
*Mixed race/other*	15%	13%	13%	8%
Highest level of education				
*Less than high school*	19%	25%	26%	16%
*High school diploma or equivalent*	35%	34%	27%	31%
*Some college/Associates degree/Technical school*	39%	36%	42%	41%
*College degree or higher*	7%	6%	6%	11%
Marital status				
*Single*	79%	84%	79%	75%
*Married*	8%	10%	7%	11%
*Divorced/Widowed*	13%	6%	14%	14%
Gestational age, *mean (SD)*	18.9(5.0)	23.3(3.5)*	16.9(5.1)*	7.8(2.3)*
Nulliparous	34%	46%*	36%	34%
No previous abortion	54%	60%	49%	53%
The abortion decision was very difficult	31%	29%	17%*	21%*
Abortion preference for man involved in pregnancy (MIP)				
*To carry pregnancy to term*	22%	26%	17%	19%
*He wasn't sure*	20%	16%	13%	20%
*To end the pregnancy*	21%	25%	31%	31%*
*He wanted her to decide*	19%	13%	19%	12%*
*MIP not involved/Don't know*	19%	20%	20%	17%
*Number of people disclosed abortion seeking (other than partner)*		*		
*No one (other than partner)*	28%	33%	33%	32%
*One person*	30%	36%	27%	28%
*Two or more people*	42%	31%	40%	41%
Who you told about abortion seeking (other than partner)				
*Parents*	34%	30%	43%	27%*
*Other family members*	34%	31%	30%	34%
*Friends*	40%	31%*	39%	43%
Emotional social support, *mean (SD)*	19.4(3.9)	19.0(3.9)	19.3(3.4)	19.4(4.0)
Ever diagnosed with anxiety or depression	27%	21%	31%	32%
Region of residence				
*West*	28%	25%	36%	26%
*Northeast*	15%	20%	7%	13%
*Midwest*	28%	28%	31%	27%
*South*	29%	28%	26%	34%
Exposure to anti-abortion protesters		*		
*Did not see (reference)*	50%	62%	57%	53%
*Saw protesters but not heard*	18%	13%	14%	13%
*Heard but was not stopped*	16%	9%	14%	16%
*Was stopped by protesters*	16%	16%	14%	18%

SD = standard deviation

* p < .05; p values are based on mixed effects regression analyses accounting for clustering by site and test for differences compared to the *Near-limit* abortion group, the reference group.

## Results

Of the 1,132 eligible people recruited, 956 people (84.5%) completed baseline interviews. Participation and attrition rates by study group have been published elsewhere [35]. We removed three people who changed their mind about wanting an abortion and 25 people who did not respond to the stigma items at baseline, leaving a final analysis sample of 928 (435 *Near-limits*, 159 *Turnaway-births*, 70 *Turnaway-no-births*, and 264 *First-trimesters*).

### Baseline perceived abortion stigma

We present participant baseline characteristics by study group in Table 1 and their baseline perceptions of abortion stigma in Table 2. According to unadjusted analyses, at approximately one-week after being denied an abortion, over half of people seeking abortion reported that people in their community (60%) or people close to them (56%) would look down on them if they knew they had sought an abortion (Table 2). People in the *Turnaway-birth* group scored significantly lower on overall and individual perceived abortion stigma items, than people in the *Near-limit* group (Table 2). African American/Black people scored lower on both stigma items when compared to White people. People who reported that the man involved in the pregnancy was not involved in the abortion decision had higher levels of perceived abortion stigma than people who reported that the man involved wanted to carry the pregnancy to term. When compared to people who did not tell anyone other than the man involved in the pregnancy that they were seeking an abortion, those who told two or more people reported significantly lower perceived abortion stigma overall (1.6 vs 1.3, p < .05) and from their community (1.6 vs 1.2, p < .05). People who were stopped by clinic protestors reported significantly higher perceptions of abortion stigma (1.7), than people who did not see protestors (1.3, p < .05). There were no significant differences in baseline perceived abortion stigma and being lost at the last interview wave five years later.

**Table 2:** Baseline perceived abortion stigma by certain participant descriptive characteristics, unadjusted (n = 928).

	Overall score Mean (SD)	Felt would be looked down upon by community (0-not at all to 4-extremely)				Felt would be looked down upon by people close to you (0-not at all to 4-extremely)			
		Mean (SD)	Not at all	A little bit/ moderately	Quite a bit/ extremely	Mean (SD)	Not at all	A little bit/ moderately	Quite a bit/ extremely
Total	1.5(1.4)	1.5(1.5)	40%	29%	31%	1.4(1.5)	44%	30%	27%
Study group									
*Near-limit (reference)*	1.6(1.4)	1.6(1.6)	38%	29%	33%	1.5(1.5)	37%	34%	29%
*First-trimester*	1.4(1.3)	1.5(1.5)	39%	31%	30%	1.3(1.5)	45%	31%	24%
*Turnaway-birth*	**1.2(1.4)**	**1.2(1.5)**	50%	26%	24%	**1.1(1.5)**	58%	24%	24%
*Turnaway-no-birth*	1.7(1.4)	1.9(1.7)	36%	24%	40%	1.5(1.6)	44%	29%	29%
Race/ethnicity									
*White*, *Non-Hispanic (reference)*	1.6(1.3)	1.8(1.5)	30%	34%	37%	1.5(1.4)	37%	37%	26%
*African American/Black*	**1.2(1.4)**	**1.2(1.5)**	52%	25%	23%	**1.2(1.5)**	52%	26%	22%
*Latina*	1.5(1.5)	1.5(1.6)	41%	25%	34%	1.5(1.6)	43%	23%	34%
*Other*	1.4(1.4)	1.5(1.6)	43%	28%	29%	1.4(1.5)	43%	30%	27%
Abortion preference for man involved in the pregnancy (MIP)									
*To carry pregnancy to term (reference)*	1.4(1.5)	1.4(1.6)	46%	26%	29%	1.3(1.5)	47%	26%	28%
*He wasn't sure*	1.3(1.3)	1.5(1.5)	40%	33%	27%	1.2(1.4)	47%	32%	21%
*To end the pregnancy*	1.5(1.4)	1.7(1.6)	37%	29%	33%	1.5(1.5)	40%	34%	27%
*He wanted her to decide*	1.3(1.3)	1.3(1.5)	47%	26%	27%	1.2(1.5)	50%	27%	23%
*MIP not involved/Don't know*	**1.7(1.4)**	**1.8(1.6)**	33%	28%	39%	**1.6(1.6)**	37%	29%	34%
Number of people disclosed abortion seeking (other than partner)									
*No one (other than partner)*	1.6(1.5)	1.7(1.6)	40%	25%	35%	1.6(1.6)	40%	26%	34%
*One person*	1.4(1.4)	1.5(1.5)	40%	25%	35%	1.4(1.5)	40%	26%	34%
*Two or more people*	**1.3(1.3)**	1.5(1.5)	40%	30%	29%	**1.2(1.4)**	45%	32%	23%
Exposure to anti-abortion protesters									
*Did not see (reference)*	1.3(1.4)	1.4(1.5)	44%	28%	28%	1.3(1.5)	49%	26%	25%
*Saw protesters but not heard*	1.5(1.4)	1.6(1.6)	42%	23%	35%	1.5(1.6)	43%	30%	27%
*Heard but was not stopped*	1.6(1.4)	1.8(1.6)	32%	33%	35%	1.4(1.5)	40%	34%	26%
*Was stopped by protesters*	**1.7(1.3)**	1.7(1.5)	34%	32%	34%	**1.7(1.5)**	28%	39%	32%
History of depression or anxiety									
*Yes*	**1.7(1.3)**	**1.9(1.5)**	29%	31%	41%	1.5(1.5)	38%	34%	28%
*No (reference)*	1.4(1.4)	1.4(1.5)	45%	28%	27%	1.3(1.5)	46%	28%	26%
Completed five-year follow-up interview									
*Yes*	1.4(1.4)	1.5(1.5)	37%	31%	32%	1.4(1.5)	42%	32%	26%
*No (reference)*	1.5(1.4)	1.6(1.5)	44%	25%	30%	1.4(1.5)	46%	27%	27%

**Bold** items are statistically significant at p < .05, based on unadjusted mixed effects regression accounting for clustering by site; SD = standard deviation.

### Five-year perceived abortion stigma trajectories

**Fig 1:**
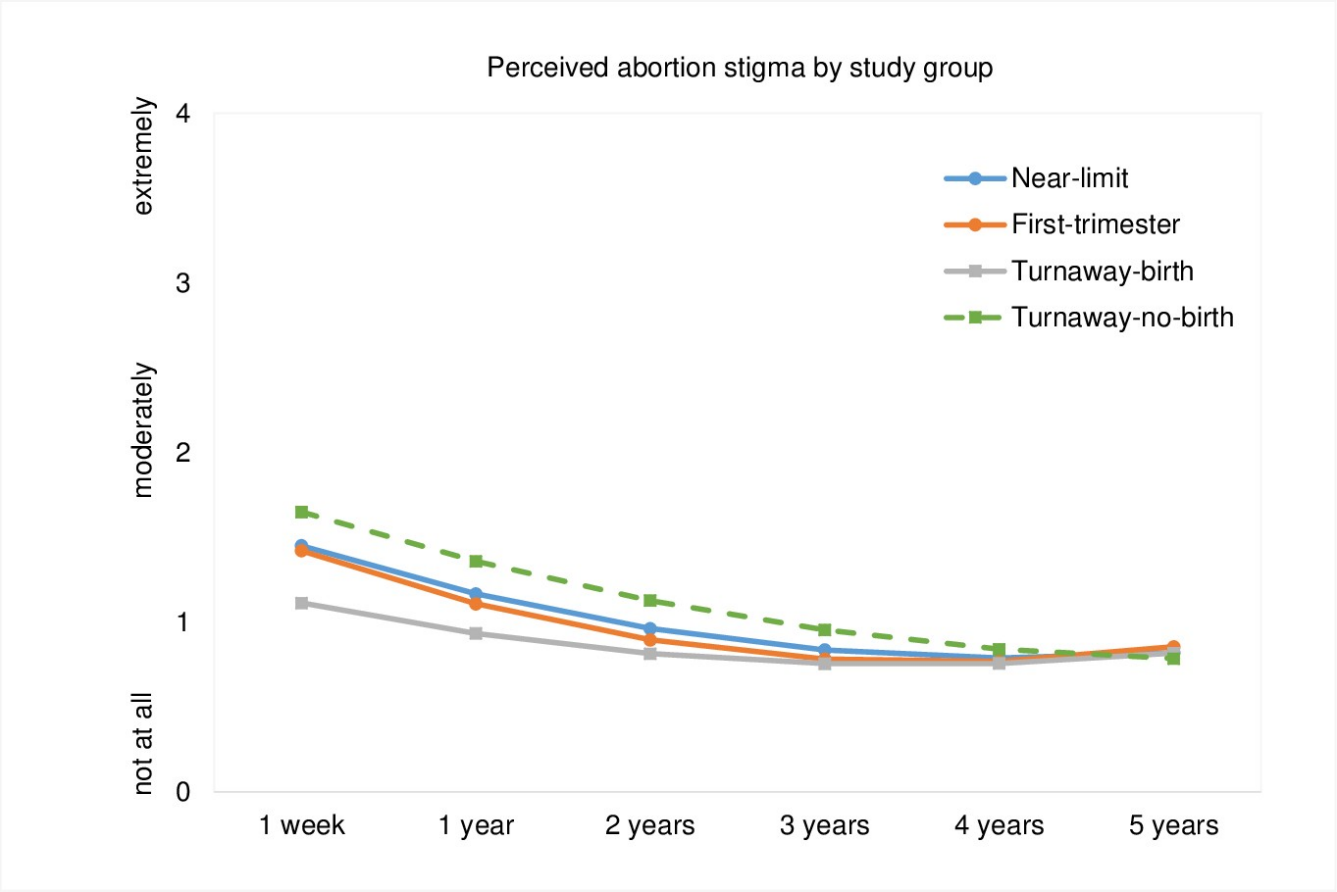
Perceived abortion stigma marginal predicted probabilities over time by study group, using adjusted mixed effects linear regression. Note: Values are based on post-estimation estimates following an adjusted mixed effects linear regression analysis controlling for race/ethnicity, age, education, marital status, parity, difficulty deciding to have an abortion, history of abortion, region of residence, religion, exposure to abortion protesters, emotional social support, and history of depression or anxiety; Differences in perceived abortion stigma between the *Turnaway-birth* and *Near-limit* abortion groups are statistically significant between one week and 1.5 years post-abortion seeking.

According to adjusted mixed effects linear regression analyses, when compared to *Near-limits*, people in the *Turnaway-birth* group reported significantly lower baseline perceived stigma from people in their community (-0.30; 95% CI, -0.52, -0.08), from people close to them (-0.38; 95% CI, -0.59, -0.16) and overall (-0.34; 95% CI, -0.54, -0.14, Table 3). People in the *Turnaway-no-birth* group were significantly more likely to perceive community abortion stigma than people in the *Near-limit* group (0.34; 95% CI, 0.03, 0.64). From one week to five years post-abortion seeking, overall perceived abortion stigma declined significantly (p < .001) for all study groups (Fig 1). These study group differences were no longer statistically significant (p>0.05) by two years post-abortion seeking (not shown).

**Table 3:** Predictors of perceived abortion stigma over five years, using adjusted mixed effects linear regression .

Independent variables	Overall perceived abortion stigma	Felt would be looked down upon by…	
		Your community	People close to you
Study group	Beta[95% CI]	Beta[95% CI]	Beta[95% CI]
*Near-limits (reference)*			
*First-trimesters*	-0.03[-0.20,0.14]	0.02[-0.16,0.21]	-0.08[-0.26,0.10]
*Turnaway-births*	**-0.34**[-0.54,-0.14]	**-0.30**[-0.52,-0.08]	**-0.38**[-0.59,-0.16]
*Turnaway-no-births*	0.20[-0.08,0.48]	**0.34**[0.03,0.64]	0.06[-0.24,0.35]
Years	**-0.32**[-0.38,-0.26]	**-0.33**[-0.40,-0.26]	**-0.32**[-0.38,-0.25]
First-trimester X years	-0.04[-0.14,0.06]	-0.08[-0.19,0.04]	-0.01[-0.12,0.10]
Turnaway-births X years	0.11[-0.00,0.23]	0.06[-0.07,0.20]	**0.16**[0.02,0.29]
Turnaway-no-births X years	0.00[-0.16,0.14]	-0.05[-0.24,0.14]	0.05[-0.14,0.24]
Years^2^	**0.04**[0.03,0.05]	**0.04**[0.03,0.05]	**0.04**[0.02,0.05]
First-Trimester X years^2^	0.01[-0.01,0.03]	0.02[-0.01,0.04]	0.01[-0.02,0.03]
Turnaway-Births X years^2^	-0.01[-0.03,0.01]	0.00[-0.03,0.03]	-0.02[-0.04,0.02]
Turnaway-No-Births X years^2^	-0.01[-0.04,0.02]	0.00[-0.04,0.04]	-0.02[-0.04,0.02]
Baseline Covariates			
Race/ethnicity:			
*White*, *Non-Hispanic (reference)*			
*African American/Black*	**-0.37**[-0.54,-0.20]	**-0.45**[-0.63,-0.27]	**-0.29**[-0.47,-0.12]
*Hispanic/Latina*	0.03[-0.16,0.22]	-0.01[-0.22,0.18]	0.09[-0.11,0.28]
*Other*	-0.02[-0.22,0.19]	-0.02[-0.24,0.20]	-0.02[-0.23,0.20]
Age			
*14–19 (reference)*			
*20–24*	**-0.19**[-0.39,-0.00]	**-0.25**[-0.45,-0.04]	-0.15[-0.35,0.04]
*25–29*	**-0.31**[-0.54,-0.09]	**-0.36**[-0.60,-0.12]	**-0.29**[-0.51,0.06]
*30–46*	**-0.26**[-0.50,-0.02]	-**0.32**[-0.58,-0.07]	-0.21[-0.46,0.03]
Highest level of education			
*Less than high school (reference)*			
*High school diploma or GED*	-0.02[-0.20,0.16]	-0.01[-0.20,0.19]	-0.04[-0.22,0.15]
*Some college/Associates/Technical school*	0.06[-0.13,0.25]	0.06[-0.14,0.26]	0.05[-0.14,0.25]
*College degree or higher*	-0.00[-0.29,0.29]	-0.02[-0.33,0.28]	0.01[-0.29,0.31]
Marital status			
*Single/Never married (reference)*			
*Married*	**0.25**[0.03,0.47]	**0.25**[0.02,0.48]	**0.26**[0.03,0.48]
*Divorced/Widowed*	0.10[-0.11,0.31]	0.16[-0.06,0.38]	0.05[-0.17,0.26]
Nulliparous	0.09[-0.06,0.25]	0.13[-0.04,0.29]	0.06[-0.10,0.22]
No previous abortion	**0.20**[0.07,0.33]	**0.18**[0.04,0.32]	**0.22**[0.09,0.36]
Difficulty deciding to have an abortion	**0.10**[0.05,0.14]	**0.10**[0.05,0.15]	**0.09**[0.05,0.14]
Emotional social support	**-0.05**[-0.06,-0.03]	**-0.04**[-0.05,-0.02]	**-0.05**[-0.06,-0.03]
Religion			
*None (reference)*			
*Protestant*	**0.20**[0.06,0.35]	**0.18**[0.02,0.34]	**0.21**[0.05,0.37]
*Catholic*	**0.28**[0.09,0.48]	**0.24**[0.04,0.45]	**0.30**[0.09,0.50]
*Other*	-0.05[-0.38,0.29]	-0.08[-0.44,0.28]	0.04[-0.31,0.39]
Exposure to anti-abortion protesters			
*Did not see (reference)*			
*Saw protesters but not heard*	0.10[-0.09,0.29]	0.09[-0.11,0.29]	0.11[-0.09,0.30]
*Heard but was not stopped*	0.04[-0.16,0.24]	0.00[0.21,0.22]	0.06[-0.15,0.27]
*Was stopped by protesters*	0.12[-0.07,0.31]	0.07[-0.13,0.27]	0.17[-0.02,0.37]
Region of residence			
*West (reference)*			
*Northeast*	-0.03[-0.24,0.18]	0.00[-0.22,0.23]	-0.03[-0.24,0.18]
*Midwest*	0.12[-0.07,0.31]	0.19[-0.01,0.40]	0.12[-0.07,0.31]
*South*	**0.29**[0.10,0.47]	**0.37**[0.17,0.57]	**0.29**[0.10,0.47]
History of depression or anxiety	**0.20**[0.06,0.35]	**0.28**[0.12,0.43]	0.12[-0.03,0.27]

**Bold** items are statistically significant at p < .05; CI = 95% confidence interval.

With the exception of education level, nulliparity and exposure to anti-abortion protesters, all covariates were significantly associated with perceived abortion stigma. When compared to White people, African American/Black people were significantly less likely to perceive abortion stigma by their community (-0.45; 95% CI, -0.63, -0.27, Table 3), people close to them (-0.29; 95% CI, -0.47, -0.12) and overall (-0.37; 95% CI, -0.54, -0.20). Married people were significantly more likely than never married people, people who identified as Protestant or Catholic religion were more likely than people who identified as no religion, and people living in the South were more likely than people living in the West to perceive abortion stigma from their community, people close to them and overall. People with no prior history of abortion and who reported difficulty deciding to have the abortion were significantly more likely than their counterparts to perceive abortion stigma from their community, from people close to them, and overall. Higher emotional social support was associated with lower perceived abortion stigma. People with a history of depression or anxiety were significantly more likely than people without such a history to perceive abortion stigma from their community and overall. When compared to people ages 19 and under, those ages 20 and over were significantly less likely to perceive abortion stigma overall and from their community, whereas people ages 25–29 were significantly less likely to perceive abortion stigma from people close to them, than people ages 19 and under.

### Perceived abortion stigma, abortion disclosure, race/ethnicity and psychological distress

**Fig 2:**
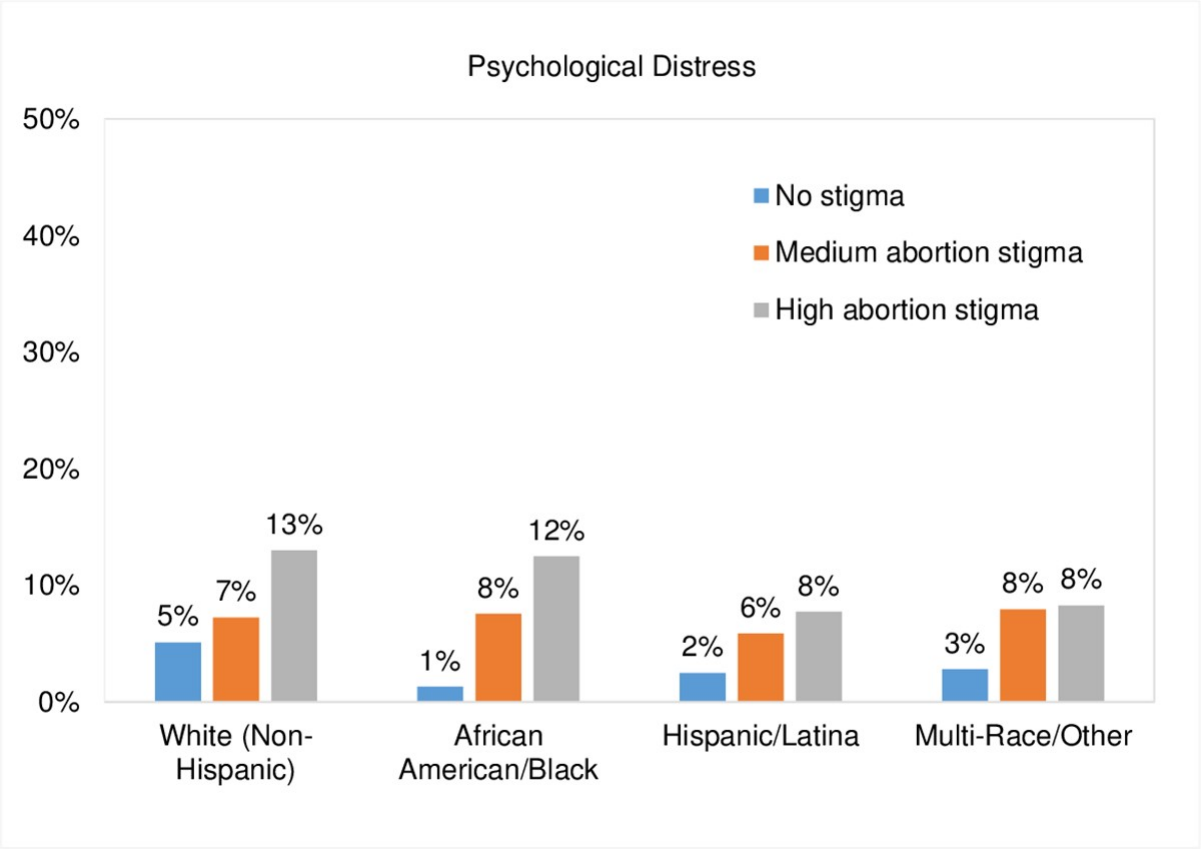
Marginal predicted probabilities of experiencing psychological distress from six months to five years post-abortion seeking by race/ethnicity and baseline perceived abortion stigma, using adjusted mixed effects logistic regression. Note: Values are based on post-estimation estimates following an adjusted mixed effects logistic regression analysis controlling for study group, number of abortion disclosures, age, education, marital status, parity, difficulty deciding to have an abortion, emotional social support, pregnancy preference of the man involved in the pregnancy, and history of depression or anxiety.

In adjusted multivariable analyses, people who reported high overall perceived abortion stigma at baseline had significantly higher odds of reporting psychological distress years later (aOR; 3.98, 95% CI, 1.39, 11.37, Table 4 and Fig 2). People who told one (aOR; 2.22, 95% CI, 1.13, 4.37) or more (aOR; 2.21, 95% CI, 1.16, 4.21) people that they had sought an abortion (other than the man involved) also had higher odds of reporting psychological distress years later than people who told no one. According to the main effects for race/ethnicity, among those without any baseline perceived abortion stigma, African American/Black people had significantly lower odds (aOR; 0.13, 95% CI, 0.03, 0.61) as non-Hispanic White people to experience psychological distress. Significant race/ethnicity by abortion stigma interaction terms indicated that race/ethnicity modified the relationship between perceptions of abortion stigma and psychological distress (Table 4 and Fig 2). As seen in Fig 2 and Table 5, among people without any reported baseline overall abortion stigma, African American/Black people had significantly lower psychological distress than non-Hispanic White people (1.3% vs 5.1%, p < .01), whereas there were no statistically significant differences in psychological distress by race/ethnicity among people with medium or high baseline perceived abortion stigma. However, while not statistically significant, among those with high perceived abortion stigma, Hispanic/Latina people were less likely to report psychological distress than non-Hispanic White people (8% vs. 13%, p = 0.056).

**Table 4:** Predictors of psychological distress over five years according to mixed effects logistic regression analyses.

	Psychological distress
Independent variables	aOR[95% Confidence Interval]
*Baseline perceived abortion stigma*	
*None (reference)*	
*Medium*	1.51[0.52,4.35]
*High*	**3.98**[1.39,11.37]
*Number of people disclosed abortion seeking (other than partner)*	
*No one (reference)*	
*One person*	**2.22**[1.13,4.37]
*Two or more people*	**2.21**[1.16,4.21]
Race/ethnicity^ǂ^	
*White*, *Non-Hispanic (reference)*	
*African American/Black*	**0.13**[0.03,0.61]
*Hispanic/Latina*	0.33[0.07,1.64]
*Multi-race/Other*	0.39[0.07,2.31]
Race/ethnicity *X baseline abortion stigma*	
*African American/Black X medium abortion stigma*	**8.11**[1.38,47.60]
*Hispanic/Latina X medium abortion stigma*	2.13[0.30,14.87]
*Multi-race/Other X medium abortion stigma*	3.01[0.34,26.73]
*African American/Black X high abortion stigma*	**6.94**[1.14,42.20]
*Hispanic/Latina X high abortion stigma*	1.14[0.18,7.34]
*Multi-race/Other X high baseline abortion stigma*	1.08[0.12,9.64]
Baseline covariates	
Study group	
Near-Limits (reference)	
First-Trimesters	1.10[0.61,1.99]
Turnaway-Births	1.17[0.55,2.51]
Turnaway-No-Births	**2.84**[1.13,7.13]
Age	
*14–19 (reference)*	
*20–24*	1.03[0.44,2.40]
*25–29*	1.33[0.53,3.41]
*30–46*	**2.72**[1.03,7.13]
Highest level of education	
*Less than high school (reference)*	
*High school diploma or equivalent*	1.03[0.50,2.12]
*Some college/Associates degree/Technical school*	0.79[0.38,1.63]
*College degree or higher*	0.76[0.25,2.35]
Marital status	
*Single/Never married (reference)*	
*Married*	2.01[0.87,4.61]
*Divorced/Widowed*	0.72[0.34,1.55]
Nulliparous	0.76[0.41,1.41]
Difficulty deciding to have an abortion	0.98[0.82,1.18]
Emotional social support	**0.84**[0.79,0.90]
Abortion preference for man involved in the pregnancy (MIP)	
*To carry pregnancy to term (reference)*	
*He wasn't sure*	0.80[0.35,1.83]
*To end the pregnancy*	0.86[0.41,1.81]
*He wanted her to decide*	0.41[0.16,1.04]
*MIP not involved/Don't know*	1.15[0.53,2.48]
History of depression or anxiety	**9.73**[5.65,17.04]

aOR = Adjusted Odds Ratio; **Bold** items are statistically significant at p < .05

ǂOdds ratios for race/ethnicity main effects present main effects of people without any baseline perceived abortion stigma.

**Table 5:** Predictive margins of experiencing any psychological distress six months to five years post-abortion seeking.

Independent variable		Predictive margins	95% Confidence Intervals		p value
Number of people told about abortion (other than partner)					
	*No one (reference)*	5.02%	3.39%	6.65%	
	*One person*	**7.92%**	5.84%	9.99%	0.021
	*Two or more people*	**7.90%**	6.09%	9.70%	0.013
Baseline perceived abortion stigma					
	None (reference)	3.67%	2.19%	5.15%	
	Medium	**7.07%**	5.32%	8.81%	0.002
	High	**10.37%**	8.00%	12.73%	0.000
Race/ethnicity by baseline perceived abortion stigma					
	No abortion stigma				
	*White (reference)*	5.11%	2.32%	7.89%	
	*African American/Black*	**1.30%**	0.12%	2.49%	0.014
	*Hispanic/Latina*	2.48%	0.18%	4.77%	0.150
	*Multi-Race/Other*	2.79%	-0.13%	5.72%	0.256
	Medium abortion stigma				
	*White (reference)*	7.22%	4.58%	9.87%	
	*African American/Black*	7.54%	4.27%	10.80%	0.883
	*Hispanic/Latina*	5.86%	2.45%	9.28%	0.540
	*Multi-Race/Other*	7.92%	2.81%	13.02%	0.810
	High abortion stigma				
	*White (reference)*	12.99%	8.98%	17.00%	
	*African American/Black*	12.48%	7.05%	17.92%	0.885
	*Hispanic/Latina*	7.71%	4.00%	11.43%	0.056
	*Multi-Race/Other*	8.27%	2.84%	13.71%	0.163

Note: Values are based on post-estimation estimates following an adjusted mixed effects logistic regression analysis controlling for study group, age, education, marital status, parity, difficulty deciding to have an abortion, emotional social support, pregnancy preference of the man involved in the pregnancy, and history of depression and anxiety; **Bold** items are statistically significant at p < .05.

## Discussion

In this study we found that over half of people seeking an abortion think that people in their community and people close to them would look down on them if they knew they had sought an abortion, a proportion similar to that reported in other studies [3]. With one in four people having an abortion in their lifetime, we find that despite its commonness, abortion is a highly stigmatized experience [36] with potential implications for future psychological health.

While we hypothesized that people denied an abortion (*Turnaway-births*) would report *higher* levels of perceived abortion stigma given that people might interpret the denial of services as a stigmatizing experience, we instead found that they reported *less* perceived abortion stigma than their counterparts, a difference that remained statistically significant for nearly two years. Presumably the lower levels of perceived abortion stigma among people denied an abortion and who carried to term, stems from their not engaging in the stigmatized behavior (obtaining an abortion), thus enabling them to ward off any feelings of shame or guilt. Interestingly, people who were denied an abortion and who later miscarried or had an abortion elsewhere (*Turnaway-no-births*) reported the highest levels of perceived community abortion stigma across study groups. These people experienced not only the structural stigma of being denied an abortion but they also were exposed to the potentially stigmatizing experience of abortion or miscarriage. This group of people was also the most likely to experience subsequent psychological distress. Their higher levels of perceived abortion stigma supports our hypothesis that abortion denial is stigmatizing. Further, it suggests that people who carry a pregnancy to term are able to avert abortion stigma by meeting the cultural ideals of motherhood by having a baby, as suggested by Kumar and colleagues [4].

The emerging public discourse condemning abortions later in pregnancy through the onslaught of legal restrictions to abortion may affect how people and their communities view abortions depending on their gestational ages. Thus, while we may have expected that people obtaining abortions earlier in pregnancy to be less likely to perceive abortion stigma than those obtaining abortions at later gestational ages, we found no differences between these two groups of people. The perceived stigma of having an abortion beyond the first trimester may change if efforts to set gestational age limits earlier in pregnancy continue to increase and to change people’s moral views about abortion at differing gestational ages.

The higher levels of abortion stigma among people who obtained an abortion persisted for nearly two years, demonstrating the long-standing effects of stigma. Participants’ abortion stigma trajectories may reflect how perceptions of stigma move through stages, according to how others’ react to the stigma and as the process of disclosing the abortion becomes easier over time [37]. The sharp decline in perceived stigma over time, not only among people who carried to term but also among people who had an abortion, suggests that many people were eventually able to liberate themselves of feelings of stigma by potentially becoming more accepting of the abortion, forgetting about the abortion, and/or engaging in experiences and social interactions that reduced perceptions of stigma. The decline in perceptions of stigma coincide with a previous Turnaway Study analysis indicating that people’s negative and positive emotions about the abortion, including feelings of guilt, and thoughts about the abortion, decreased significantly over time [12].

While previous Turnaway Study findings have demonstrated that people who had abortions were not more likely to experience adverse mental health outcomes than those denied an abortion and carried their pregnancies to term, it also found that for all groups of people, psychological distress was highest at the time of seeking abortion when compared to years later [35]. In the current analysis, we found that in addition to history of mental health problems and other covariates, the higher levels of perceived abortion stigma at the time of seeking an abortion, also contribute to people’s experiences of psychological distress. Perceived abortion stigma was strongly associated with experiences of psychological distress years later, both among people who had abortions and people who were denied abortions. This association suggests that people may have internalized these perceptions of stigma, with long-term consequences for their mental health. Another interpretation to this finding is that, people may have experienced enacted stigma rather than merely perceptions of stigma, for example if they received negative reactions from others post-abortion seeking and these experiences may have led to subsequent negative mental health outcomes.

Consistent with the published literature, we found that people’s social and cultural contexts, including race/ethnicity, age, religion, and where they lived, were significantly associated with perceived abortion stigma [3, 29, 36]. One of the strengths of our study design lies that we collected data from people seeking abortions in 21 states, representing every region of the United States, which allowed us to capture the experience of a diverse range of individuals. Perceptions of stigma were higher in certain parts of the country where abortion is more restricted and among people who identified as Protestant or Catholic. Married people reported higher levels of perceived stigma than never married people. This is perhaps a product of the social pressures and expectations that married people may feel to have children, whereas unmarried people may feel more social pressure to avoid childbearing.

Of note, was the significant interaction we found by race/ethnicity and perceived abortion stigma on psychological distress. Consistent with the literature [1, 3], African American/Black people were less likely to report perceived abortion stigma compared to non-Hispanic White people. The concept of stratified reproduction, which describes the notion that society places differential valuations on reproduction, fertility, and parenthood based on race, provides some context to this finding [1, 14]. Historical injustices, including forced sterilization among mostly low-income people and people of color, and current societal practices, including preferentially encouraging long-acting reversible contraception among communities of color and low-income people, are just two examples of policies and practices that exemplify how reproduction is stratified in the United States with a devaluation of the fertility and parenthood of African American/Black people [14, 38]. In the context of abortion stigma, White people who have an abortion may experience stigma for not conforming to these societal ideals that place a greater value on their birth and parenthood, while African American/Black people may perceive less stigma from a society that devalues their childbearing [14, 39]. Our finding that race/ethnicity modifies the relationship between perceived abortion stigma and psychological distress, is new to the literature. Differences in perceived stigma levels and in the effect of stigma on psychological distress may reflect the unique types of racialized abortion stigma that people of different races face [14]. The anti-choice movement has developed targeted anti-abortion messaging that takes advantage of historical and current reproductive injustices and stratified reproduction to place blame and shame on individual African American/Black people who choose to have abortions [40]. If African American/Black people who do perceive higher baseline abortion stigma internalize racialized abortion stigma, this may carry more weight and affect psychological distress related to their abortion.

In contrast to previous studies suggesting that concealing abortion may result in negative psychological consequences, we found that telling others about one’s abortion-seeking behavior increased people’s risk for experiencing adverse psychological outcomes, while having emotional social support, as measured by the perceived availability of friends or family to talk about problems according to a validated scale [33], was protective. This finding suggests that people may have told others who reacted negatively or who were not supportive of their pregnancy decisions, thereby affecting their mental health. Another explanation for this finding is that people who felt psychologically distressed may have been more likely to disclose their abortion to others as a way to cope with their distress. Consistent with this hypothesis, a previous study found that people who experienced intrusive thoughts about the abortion were more likely to disclose their emotions about the abortion to others, which was associated with psychological distress [24]. Unfortunately our measure of telling others about the abortion is an imperfect proxy for abortion concealment, as it does not allow us to assess the extent to which people’s abortion disclosures were by necessity or by choice or how many people they may have kept the abortion secret from. We also do not know people’s reasons for disclosing and how others reacted to their disclosures.

This study has a number of limitations. Our measure of abortion disclosure was limited, in that it captured the number but not the context or consequence of disclosures. A better measure of abortion disclosure would have also revealed people’s reason for disclosing, whether they wanted to disclose or felt forced to disclose by circumstances, as well as people’s reactions to the disclosure. Thus, our findings on the relationship between abortion disclosure and psychological health, should be viewed cautiously. While our measure on perceived abortion stigma was drawn from a previously used measure [24], it was adapted to also work with people who had sought, but not necessarily obtained an abortion, limiting our ability to compare our results across studies. Neither the original nor our adapted measure have been validated, and this measure does not capture the full range of dimensions that have been described in other measures of abortion stigma [41]. However, a notable strength of our study is its longitudinal design and ability to examine the perceived abortion stigma trajectories and the process of stigmatization and de-stigmatization over five years. We are unaware of any other study that has examined perceived abortion stigma over time. The longitudinal design offers a new contribution to the literature by measuring perceived abortion stigma before our measure of psychological distress, which is an essential first step to establishing causation. Another important study strength is that we were able to compare the experiences of abortion stigma among people seeking abortion, denied an abortion, and among people seeking abortion earlier and later in pregnancy. Taken together, this study finds that people who have obtained or have considered abortion are at risk of perceiving abortion stigma which is influenced by an array of contextual factors such as race/ethnicity, religion, and social support, all of which may have consequences for their psychological well-being years later.
